# The genome sequence of the white admiral, *Limenitis camilla *(Linnaeus, 1764)

**DOI:** 10.12688/wellcomeopenres.18594.1

**Published:** 2022-12-12

**Authors:** Roger Vila, Konrad Lohse, Alex Hayward, Dominik R. Laetsch, Wellcome Sanger Institute Tree of Life programme, Wellcome Sanger Institute Scientific Operations: DNA Pipelines collective, Tree of Life Core Informatics collective, Niklas Wahlberg, Darwin Tree of Life Consortium

**Affiliations:** 1 Institut de Biologia Evolutiva, CSIC - Universitat Pompeu Fabra, Barcelona, Spain; 2 Institute of Ecology and Evolution, University of Edinburgh, Edinburgh, UK; 3 College of Life and Environmental Sciences, Department of Biosciences, University of Exeter, Exeter, UK; 4 Department of Biology, Lund University, Lund, Sweden

**Keywords:** Limenitis camilla, white admiral, genome sequence, chromosomal, Lepidoptera

## Abstract

We present a genome assembly from an individual female *Limenitis camilla* (the white admiral; Arthropoda; Insecta; Lepidoptera; Nymphalidae). The genome sequence is 435 megabases in span. Most of the assembly (99.97%) is scaffolded into 31 chromosomal pseudomolecules, corresponding to 29 autosomes plus the W and Z sex chromosomes. The complete mitochondrial genome was also assembled and is 15.2 kilobases in length. Gene annotation of this assembly on Ensembl identified 12,489 protein coding genes.

## Species taxonomy

Eukaryota; Metazoa; Ecdysozoa; Arthropoda; Hexapoda; Insecta; Pterygota; Neoptera; Endopterygota; Lepidoptera; Glossata; Ditrysia; Papilionoidea; Nymphalidae; Limenitidinae; Limenitidini; *Limenitis*; *Limenitis camilla* (Linnaeus, 1764) (NCBI:txid270466).

## Background

The white admiral, *Limenitis camilla* (Linnaeus 1764), is a widespread species in temperate Eurasia, and is found in southern Britain. While *L. camilla* is considered a species of Least Concern according to the IUCN Red List for Europe ( van Swaay *et al.*, 2010), it is listed as vulnerable on the UK Red List ( Fox *et al.*, 2022). In Britain there has been a dramatic decline in populations during the last two decades, but the reasons for this are unclear ( Fox *et al.*, 2015; Fox *et al.*, 2022).

The species is found in shady woodland areas, where its larval host plants, honeysuckles *(Lonicera* sp.), grow. Adults are attracted to bramble flowers for nectar. The white admiral is generally univoltine, but it can have two overlapping generations in some parts of its range, where it can be found as adult from May to September or even beginning of October ( Tshikolovets, 2011; Vila *et al.*, 2018).

*L. camilla* belongs to a species-rich genus that has its centre of diversity in eastern Asia ( Tseng *et al.*, 2022). Only three species are found in Europe, *L. camilla*, *L. reducta*, and *L. populi*. These three species are not closely related to each other and appear to represent independent colonisations of Europe from Asia ( Tseng *et al.*, 2022). While Lorkovic (1941) reports 31 chromosome pairs for *Limenitis camilla*, other researchers have documented 30 chromosome pairs ( Beliajeff, 1930; Maeki & Makino, 1953; Maeki, 1961).

## Genome sequence report

**Figure 1.**
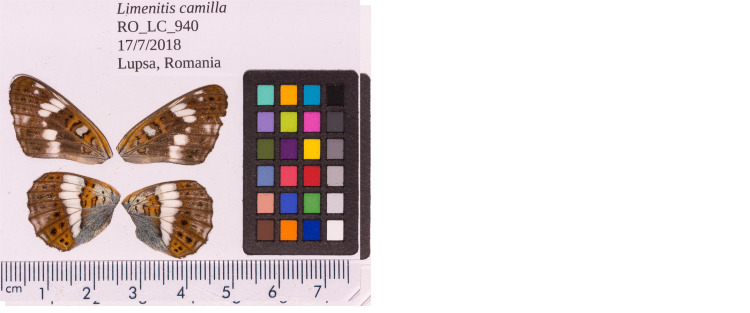
Forewings and hindwings of the female *L. camilla* specimen from which the genome was sequenced. Dorsal (left) and ventral (right) surface view of wings from specimen RO_LC_940 (ilLimCami1) from Lupşa, Alba, Romania, used to generate Pacific Biosciences, 10X genomics, Hi-C and RNA-Seq data.

The genome was sequenced from a single female *L. camilla* ( Figure 1) collected from Lupşa, Apuseni Mountains, Alba, Romania. A total of 60-fold coverage in Pacific Biosciences single-molecule HiFi long reads and 87-fold coverage in 10X Genomics read clouds were generated. Primary assembly contigs were scaffolded with chromosome conformation Hi-C data. Manual assembly curation corrected 21 missing/misjoins and removed two haplotypic duplications, reducing the assembly size by 0.01% and the scaffold number by 17.05%.

**Figure 2.**
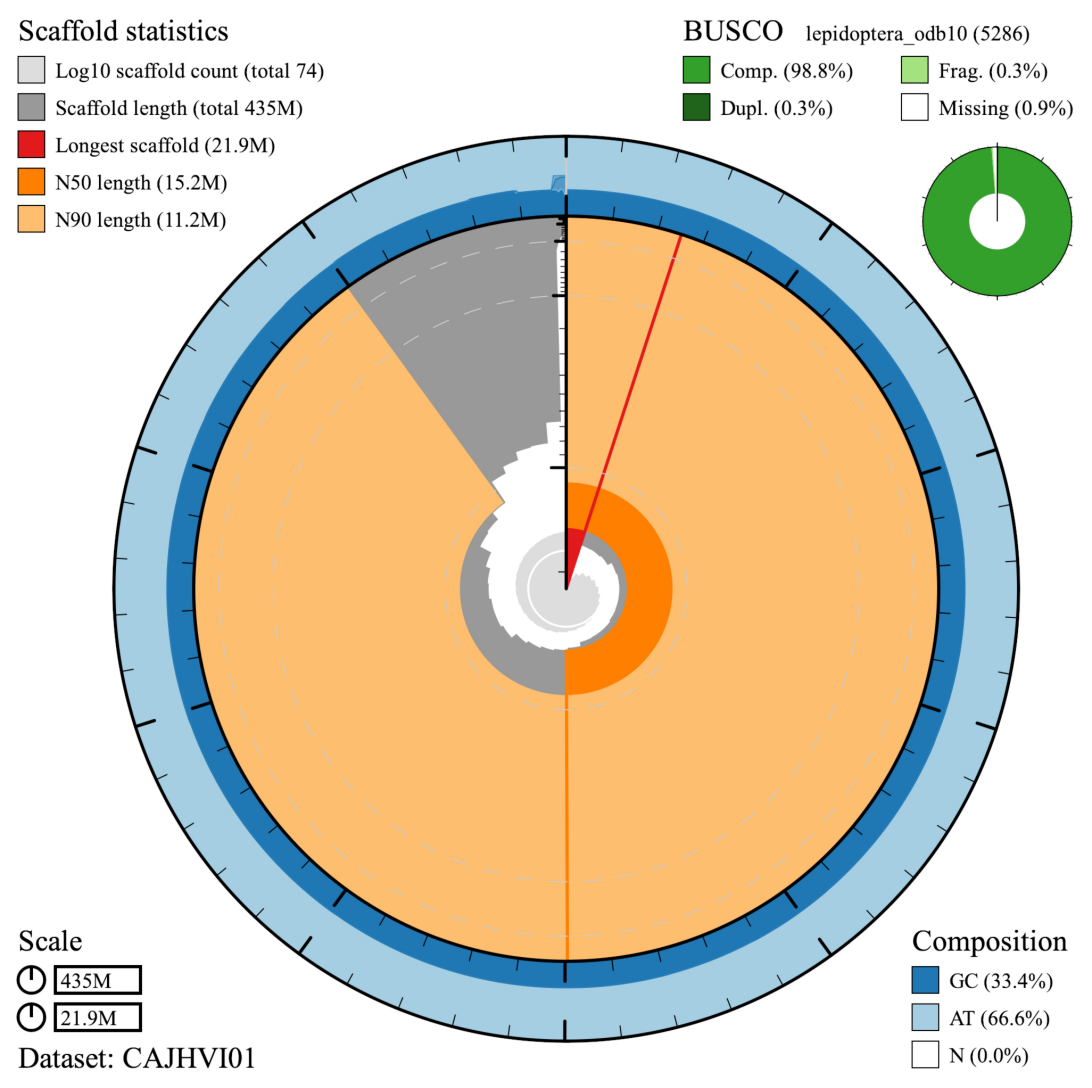
Genome assembly of *L. camilla*, ilLimCami1.1: metrics. The BlobToolKit Snailplot shows N50 metrics and BUSCO gene completeness. The main plot is divided into 1,000 size-ordered bins around the circumference with each bin representing 0.1% of the 435,112,716 bp assembly. The distribution of chromosome lengths is shown in dark grey with the plot radius scaled to the longest chromosome present in the assembly (21,933,589 bp, shown in red). Orange and pale-orange arcs show the N50 and N90 chromosome lengths (15,214,206 and 11,199,090 bp), respectively. The pale grey spiral shows the cumulative chromosome count on a log scale with white scale lines showing successive orders of magnitude. The blue and pale-blue area around the outside of the plot shows the distribution of GC, AT and N percentages in the same bins as the inner plot. A summary of complete, fragmented, duplicated and missing BUSCO genes in the lepidoptera_odb10 set is shown in the top right. An interactive version of this figure is available at https://blobtoolkit.genomehubs.org/view/ilLimCami1.1/dataset/CAJHVI01/snail.

The final assembly has a total length of 435 Mb in 73 sequence scaffolds with a scaffold N50 of 15.2 Mb ( Table 1). Most of the assembly sequence (99.97%) was assigned to 31 chromosomal-level scaffolds, representing 29 autosomes (numbered by sequence length) plus the W and Z sex chromosomes ( Figure 2– Figure 5; Table 2), in agreement with previous authors suggesting a haploid chromosome number of *n* = 30 (e.g. Beliajeff, 1930).

**Table 1.** Genome data for *L. camilla*, ilLimCami1.1.

*Project accession data*	
Assembly identifier	ilLimCami1.1
Species	*Limenitis camilla*
Specimen	ilLimCami1 (genome assembly, Hi-C, RNA-Seq)
NCBI taxonomy ID	270466
BioProject	PRJEB42132
BioSample ID	SAMEA7523310
Isolate information	Female, whole organism (ilLimCami1)
*Raw data accessions*	
PacificBiosciences SEQUEL II	ERR6576314
10X Genomics Illumina	ERR6002660-ERR6002662; ERR6003043
Hi-C Illumina	ERR6002663–ERR6002666
PolyA RNA-Seq Illumina	ERR6286705
*Genome assembly*	
Assembly accession	GCA_905147385.1
*Accession of alternate haplotype*	GCA_905147315.1
Span (Mb)	435
Number of contigs	101
Contig N50 length (Mb)	12.0
Number of scaffolds	73
Scaffold N50 length (Mb)	15.2
Longest scaffold (Mb)	16.75
BUSCO * genome score	C:98.8%[S:98.4%,D:0.3%],F:0.3%,M:0.9%,n:5,286
*Genome annotation*	
Number of protein-coding genes	12,489
Average length of coding sequence	14,179.79
Average length of coding exon size	1,531.93
Average length of coding intron size	1,694.97

*BUSCO scores based on the lepidoptera_odb10 BUSCO set using v5.3.2. C = complete [S = single copy, D = duplicated], F = fragmented, M = missing, n = number of orthologues in comparison. A full set of BUSCO scores is available at https://blobtoolkit.genomehubs.org/view/ilLimCami1.1/dataset/CAJHVI01/busco.

**Figure 3.**
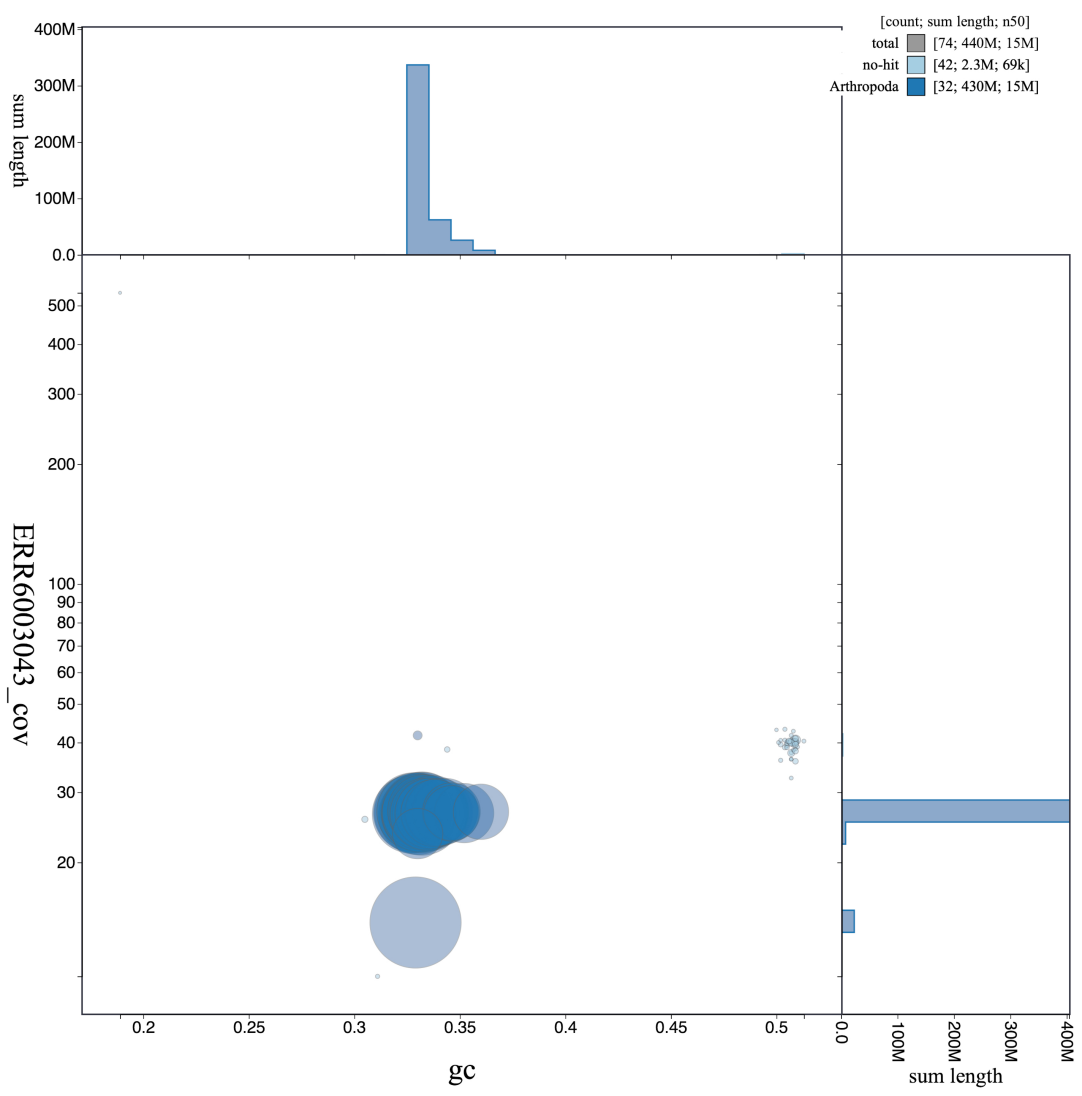
Genome assembly of *L. camilla*, ilLimCami1.1: GC coverage. BlobToolKit GC-coverage plot. Scaffolds are coloured by phylum. Circles are sized in proportion to scaffold length. Histograms show the distribution of scaffold length sum along each axis. An interactive version of this figure is available at https://blobtoolkit.genomehubs.org/view/ilLimCami1.1/dataset/CAJHVI01/blob.

**Figure 4.**
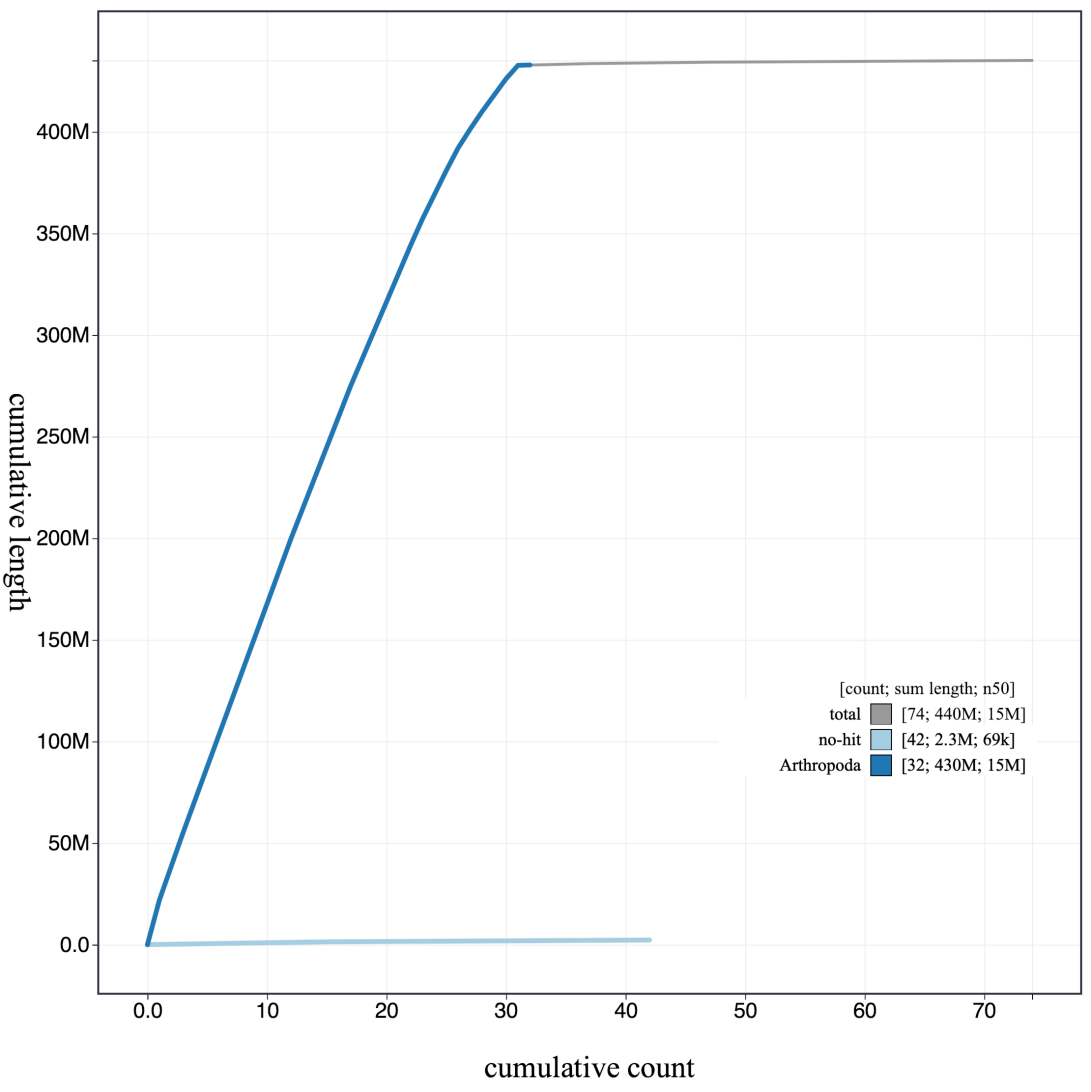
Genome assembly of *L. camilla*, ilLimCami1.1: cumulative sequence. BlobToolKit cumulative sequence plot. The grey line shows cumulative length for all scaffolds. Coloured lines show cumulative lengths of scaffolds assigned to each phylum using the buscogenes taxrule. An interactive version of this figure is available at https://blobtoolkit.genomehubs.org/view/ilLimCami1.1/dataset/CAJHVI01/cumulative.

**Figure 5.**
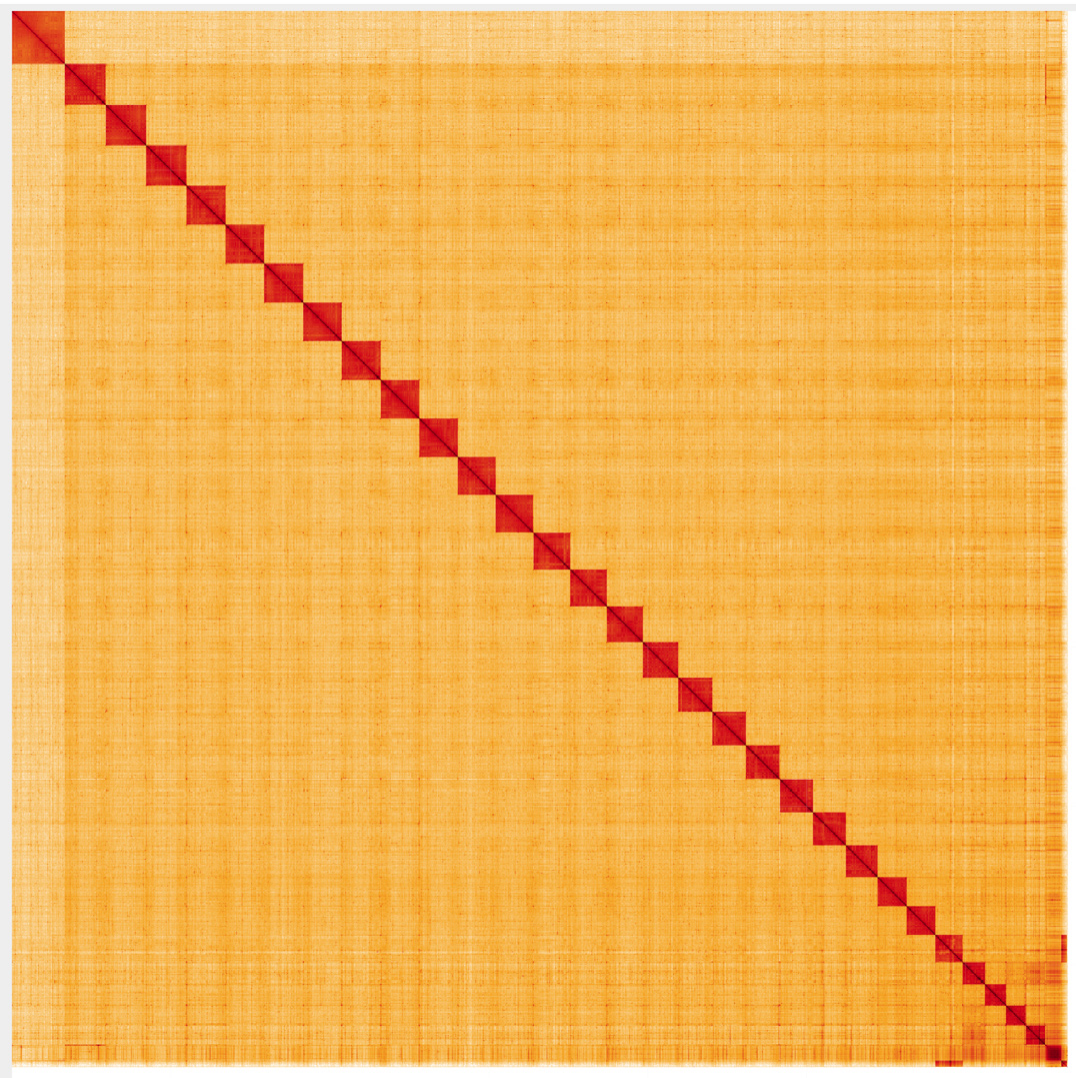
Genome assembly of *L. camilla*, ilLimCami1.1: Hi-C contact map. Hi-C contact map of the ilLimCami1.1 assembly, visualised in HiGlass. Chromosomes are arranged in size order from left to right and top to bottom. The interactive Hi-C map can be viewed at https://genome-note-higlass.tol.sanger.ac.uk/l/?d=C4aYtOqlRRioeJ532sWPiA.

**Table 2.** Chromosomal pseudomolecules in the genome assembly of *L. camilla*, ilLimCami1.1.

INSDC accession	Chromosome	Size (Mb)	GC%
LR990225.1	1	16.75	33.2
LR990226.1	2	16.71	32.8
LR990227.1	3	16.58	33.2
LR990228.1	4	16.13	33
LR990229.1	5	16.03	33.1
LR990230.1	6	16.03	32.8
LR990231.1	7	16.01	32.7
LR990232.1	8	15.98	33.1
LR990233.1	9	15.86	33.1
LR990234.1	10	15.85	33.2
LR990235.1	11	15.81	32.9
LR990236.1	12	15.43	33.2
LR990237.1	13	15.21	33.1
LR990238.1	14	15.08	33.1
LR990239.1	15	14.89	33.1
LR990240.1	16	14.63	33.4
LR990241.1	17	14.1	33.4
LR990242.1	18	13.86	33.3
LR990243.1	19	13.84	33.7
LR990244.1	20	13.66	33.5
LR990245.1	21	13.55	33.4
LR990249.1	22	11.2	34.3
LR990246.1	23	13.13	33.6
LR990247.1	24	12.01	34
LR990248.1	25	11.89	33.8
LR990250.1	26	9.21	35.2
LR990251.1	27	8.67	34.6
LR990252.1	28	8.07	34.6
LR990253.1	29	8	36
LR990254.1	W	6.6	33
LR990224.1	Z	21.93	32.9
LR990255.1	MT	0.02	19.3
-	Unplaced	2.42	48

The assembly has a BUSCO v5.3.2 ( Manni *et al.*, 2021) completeness of 98.8% (single 98.4%, duplicated 0.4%) using the lepidoptera_odb10 reference set ( *n* = 5,286). While not fully phased, the assembly deposited is of one haplotype. Contigs corresponding to the second haplotype have also been deposited.

*Genome annotation report*

Annotation of the GCA_905147385.1 assembly was generated using the Ensembl genome annotation pipeline ( Table 1; Ensembl annotation). The resulting annotation includes 12,489 protein coding genes with an average length of 14,179.79 and an average coding length of 1531.93, and 2,538 non-protein coding genes. There is an average of 7.54 exons and 6.54 introns per canonical protein coding transcript, with an average intron length of 1694.97. A total of 4763 gene loci have more than one associated transcript. The annotation has a BUSCO v5.1.2 completeness of C:96.3%[S:95.8%,D:0.5%],F:0.9%,M:2.8%,n:52,86 using lepidoptera_odb10. The annotation identified a repeat content of 38.17%.

## Methods

### Sample acquisition and nucleic acid extraction

A single female *L. camilla* specimen (ilLimCami1) was collected from Lupşa, Apuseni Mountains, Alba, Romania (latitude 46.416, longitude 23.192) by Roger Vila (Institut de Biologia Evolutiva, Barcelona), Konrad Lohse, Dominik Laetsch (both University of Edinburgh) and Alex Hayward (University of Exeter), using a handnet. The specimen was identified by Roger Vila and snap-frozen from live in a dry shipper. 

DNA was extracted at the Scientific Operations Core, Wellcome Sanger Institute. The ilLimCami1 sample was weighed and dissected on dry ice with tissue set aside for Hi-C sequencing. Whole organism tissue was disrupted by manual grinding with a disposable pestle. Fragment size analysis of 0.01–0.5 ng of DNA was then performed using an Agilent FemtoPulse. High molecular weight (HMW) DNA was extracted using the Qiagen MagAttract HMW DNA extraction kit. Low molecular weight DNA was removed from a 200-ng aliquot of extracted DNA using 0.8X AMpure XP purification kit prior to 10X Chromium sequencing; a minimum of 50 ng DNA was submitted for 10X sequencing. HMW DNA was sheared into an average fragment size of 12–20 kb in a Megaruptor 3 system with speed setting 30. Sheared DNA was purified by solid-phase reversible immobilisation using AMPure PB beads with a 1.8X ratio of beads to sample to remove the shorter fragments and concentrate the DNA sample. The concentration of the sheared and purified DNA was assessed using a Nanodrop spectrophotometer and Qubit Fluorometer and Qubit dsDNA High Sensitivity Assay kit. Fragment size distribution was evaluated by running the sample on the FemtoPulse system.

RNA was extracted from remaining whole organism tissue of ilLimCami1 in the Tree of Life Laboratory at the WSI using TRIzol, according to the manufacturer’s instructions. RNA was then eluted in 50 μl RNAse-free water and its concentration RNA assessed using a Nanodrop spectrophotometer and Qubit Fluorometer using the Qubit RNA Broad-Range (BR) Assay kit. Analysis of the integrity of the RNA was done using Agilent RNA 6000 Pico Kit and Eukaryotic Total RNA assay.

### Sequencing

Pacific Biosciences HiFi circular consensus and 10X Genomics Chromium read cloud sequencing libraries were constructed according to the manufacturers’ instructions. Sequencing was performed by the Scientific Operations core at the Wellcome Sanger Institute on Pacific Biosciences SEQUEL II (HiFi), Illumina HiSeq 10X and Illumina HiSeq 4000 (RNA-Seq) instruments. Hi-C data were generated in the Tree of Life laboratory from the remaining whole organism tissue of ilLimCami1 using the Arima v1 kit and sequenced on a HiSeq 10X instrument. 

### Genome assembly

Assembly was carried out with Hifiasm ( Cheng *et al.*, 2021); haplotypic duplication was identified and removed with purge_dups ( Guan *et al.*, 2020). One round of polishing was performed by aligning 10X Genomics read data to the assembly with longranger align, calling variants with freebayes ( Garrison & Marth, 2012). The assembly was then scaffolded with Hi-C data ( Rao *et al.*, 2014) using SALSA2 ( Ghurye *et al.*, 2019). The assembly was checked for contamination and corrected using the gEVAL system ( Chow *et al.*, 2016) as described previously ( Howe *et al.*, 2021). Manual curation ( Howe *et al.*, 2021) was performed using gEVAL, HiGlass ( Kerpedjiev *et al.*, 2018) and Pretext ( Harry, 2022). The mitochondrial genome was assembled using MitoHiFi ( Uliano-Silva *et al.*, 2021), which performs annotation using MitoFinder ( Allio *et al.*, 2020). The genome was analysed and BUSCO scores were generated within the BlobToolKit environment ( Challis *et al.*, 2020). Table 3 contains a list of all software tool versions used, where appropriate.

**Table 3.** Software tools used.

Software tool	Version	Source
BlobToolKit	3.2.6	( Challis *et al.*, 2020)
freebayes	1.3.1-17-gaa2ace8	( Garrison & Marth, 2012)
gEVAL	N/A	( Chow *et al.*, 2016)
Hifiasm	0.12	( Cheng *et al.*, 2021)
HiGlass	1.11.6	( Kerpedjiev *et al.*, 2018)
longranger align	2.2.2	https://support.10xgenomics.com/genome-exome/ software/pipelines/latest/advanced/other-pipelines
MitoHiFi	1.0	( Uliano-Silva *et al.*, 2021)
PretextView	0.2.x	https://github.com/wtsi-hpag/PretextView
purge_dups	1.2.3	( Guan *et al.*, 2020)
SALSA2	2.2	( Ghurye *et al.*, 2019)

### Genome annotation

The Ensembl gene annotation system ( Aken *et al.*, 2016) was used to generate annotation for the *L. camilla* assembly (GCA_905147385.1). Annotation was created primarily through alignment of transcriptomic data to the genome, with gap filling via protein-to-genome alignments of a select set of proteins from UniProt ( UniProt Consortium, 2019).

## Data Availability

European Nucleotide Archive: *Limenitis camilla* (white admiral). Accession number PRJEB42132; https://identifiers.org/ena.embl/PRJEB42132 ( Wellcome Sanger Institute, 2022). The genome sequence is released openly for reuse. The *L. camilla* genome sequencing initiative is part of the Darwin Tree of Life (DToL) project. All raw sequence data and the assembly have been deposited in INSDC databases. Raw data and assembly accession identifiers are reported in Table 1.

## Author information

Members of the Wellcome Sanger Institute Tree of Life programme are listed here: https://doi.org/10.5281/zenodo.4783585.

Members of Wellcome Sanger Institute Scientific Operations: DNA Pipelines collective are listed here: https://doi.org/10.5281/zenodo.4790455.

Members of the Tree of Life Core Informatics collective are listed here: https://doi.org/10.5281/zenodo.5013541.

Members of the Darwin Tree of Life Consortium are listed here: https://doi.org/10.5281/zenodo.4783558.
