# Supplementary material for: The genome sequence of the white admiral, Limenitis camilla (Linnaeus, 1764)
Source: Wellcome Open Res. 2022 Dec 12;7:301. [Version 1] doi: 10.12688/wellcomeopenres.18594.1 (PMC10539722; doi:10.12688/wellcomeopenres.18594.1)
Supplement: Peer review history

# Reviewer report: Reviewer response for version 1

**DOI:** 10.21956/wellcomeopenres.20619.r67052

**Published:** 2023-09-28

**Authors:** Kuppusamy Sivasankaran

**Affiliations:** 1 Division of Taxonomy and Biodiversity, Entomology Research Institute, Loyola College, Chennai, Tamil Nadu, India

**Review report**

 I admire the author for compiling *Limenitis camilla's* (Linnaeus 1764) whole genomic sequence. The authors have followed the appropriate techniques for whole genome sequencing. The proper software was followed for genome assembly and sequence annotation. The authors have annotated PCGs in the whole genome sequence. They have given the total number of PCGs (12,489) in the table.

**The few comments on the manuscripts**

**Comment 1:**  According to Fox *et al.,* 2022 the species *Limenitis camilla* (Linnaeus 1764) is in the vulnerable list. Have the authors got approval from the Insect Red Data Book Committee or Royal Society for Nature Conservation, UK for DNA isolation?

**Comment 2:** Authors have assembled the mitochondrial genome in MitoHiFi software. But they haven’t given the total length of mitochondrial genome sequence in the text. The mitochondrial genome length could be added in the text.

 Overall, the manuscript can be accepted for indexing.

Are sufficient details of methods and materials provided to allow replication by others?

Yes

Is the rationale for creating the dataset(s) clearly described?

Yes

Are the datasets clearly presented in a useable and accessible format?

Yes

Are the protocols appropriate and is the work technically sound?

Yes

Reviewer Expertise:

Phylogenetic analysis of clade Macroheterocera moths (Lepidoptera) using mitochondrial genome sequence.

I confirm that I have read this submission and believe that I have an appropriate level of expertise to confirm that it is of an acceptable scientific standard.

# Reviewer report: Reviewer response for version 1

**DOI:** 10.21956/wellcomeopenres.20619.r67056

**Published:** 2023-09-28

**Authors:** Elena A Pazhenkova

**Affiliations:** 1 Department of Biology, Biotechnical Faculty, University of Ljubljana, Ljubljana, Slovenia

This article presents the genome assembly of the white admiral butterfly, *Limenitis camilla*. The authors have successfully employed state-of-the-art sequencing technologies and assembly tools, resulting in a comprehensive genomic resource. The assembled genome, spanning 435 megabases, is scaffolded into 31 chromosomal pseudomolecules (including 30 autosomes, W and Z chromosomes) provides a clear representation of the genome structure.

 The gene annotation, conducted using Ensembl, identifies a substantial number of protein coding genes, contributing significantly to our understanding of the genetic basis of *L. camilla.* The methods section is detailed and well-structured, covering sample acquisition, nucleic acid extraction, and sequencing, ensuring the reliability of the obtained genomic data.

 Overall, this article stands as a valuable contribution to the field of biodiversity research and genomics. The genome assembly of *Limenitis camilla*, along with the ecological context provided, opens avenues for further investigations into the species' biology, adaptation, and population genomics.

Are sufficient details of methods and materials provided to allow replication by others?

Yes

Is the rationale for creating the dataset(s) clearly described?

Yes

Are the datasets clearly presented in a useable and accessible format?

Yes

Are the protocols appropriate and is the work technically sound?

Yes

Reviewer Expertise:

Bioinformatics, entomology

I confirm that I have read this submission and believe that I have an appropriate level of expertise to confirm that it is of an acceptable scientific standard.

# Reviewer report: Reviewer response for version 1

**DOI:** 10.21956/wellcomeopenres.20619.r53865

**Published:** 2023-01-20

**Authors:** Julie C Dunning Hotopp

**Affiliations:** 1 Institute for Genome Sciences, Health Sciences Facility III, University of Maryland School of Medicine, Baltimore, MD, USA

This manuscript by Vila et al. describes "The genome sequence of the white admiral, *Limenitis camilla*". I enjoyed reading this announcement. Thanks for the work you have done on this. I apologize that my review is probably longer than your data note. I think most of these things can be addressed with just a few words, but I felt I owed an explanation for why I was asking, which contributed to the length of the review.

 In the introduction, it would be helpful to know what type of chromosomes this species has with respect to metacentric vs. holocentric vs. telocentric. I suspect they should have telomeres, although biological diversity is great and I try not to make assumptions. I don't see telomeres mentioned at all; I am curious if you were able to use them to orient contigs in the pseudomolecule or if they were unresolved.

 Figure 2, I don't understand most of the image (gray/peach/orange portions).  The data presented in the corners are odd because they could just be put in Table 1. The blue and pale blue make sense, although they are not informative since there is little to no variation from the mean. This likely owes to the size of the bins and/or the scale. The size of the bins is also not clear to me.  Are there 1000 of them, presumably each 435,113 bp in size? Or are they each 1000 bp? Either way the bin size is too large to be informative about events that would alter GC content like the presence of ncRNAs or as a proxy for atypical nucleotide content. Most of the information in this figure (possibly all of the information in this figure) would be better served being moved to Table 1 and removing the figure. To beat a dead horse (in case you leave the figure in), I also don't understand what the two scales are. The 400+ Mbp  one makes sense. But what is the other?

 Figure 3, the coloring in the key doesn't match the coloring in the figures. I'm curious about what is going on with the contigs at 50% GC that don't get mentioned. Are they the rRNA arrays? A virus (integrated or otherwise)? Some sort of gene transfer event? I was initially guessing that the large circle that is at 1/2 sequencing depth is probably a sex chromosome but it is hard to tell if it matches Arthropods or something else. I'm also unsure in a female from an W/Z system if you expect to have 1/2 sequencing depth for one or both of the sex chromosomes. You need to explain what the expected chromosomal state of a female in this particular insect (since it can vary even in XY and XO systems, with which most people are familiar). But back to the coloring, I can't tell if these sequences are arthropod or not.  This is true for the static image as well as when you open the blobtoolkit URL. I would suggest that since there seems to be some use of transparency in rendering the figure, you end up with shading. Because of this, you likely need to use two different colors, not two shades of the same/similar color. Regardless, a discussion on contamination and contamination removal is needed. Did you have absolutely no contamination. Do you think any of these outliers are contamination? This feeds into the question at the end about Wolbachia and endosymbionts as well.

 The same coloring problem exists for Figure 4. In addition, I question the inclusion of Figure 4 as I don't see what value it adds.

 For table 2, it would be helpful to have the sequencing depth for the various technologies in this table. It would also be helpful to know how many contigs and/or scaffolds are in each pseudomolecule as well as how many telomeres and/or centromeres (if they even have centromeres, see previous comment on this). 

 I am so glad you included Figure 5, but it really needs to be labeled with the pseudomolecule numbers. It seems obvious until you try to figure out where Z and W are. If Z and W were labeled I would know if that unusual sharing between some of the last pseudomolecules are potential misassemblies or something like a pseudoautosomal region. (A sentence or two discussing this would be helpful). Related to that, it would be helpful if you mention if you even expect a pseudoautosomal region. I know they happen in XY systems, so I assume they do in ZW systems, but biology is incredibly rich in diversity.

 Fragment size analysis is described before DNA extraction, which seems odd to me. Please clarify, particularly since fragment size is described twice. I'm unclear what was done.

 How was the Illumina RNAseq library prepared (e.g. what kit and/or reagents was/were used)? Was it really only from the one female? (If so, are you only getting female transcripts?) How were the reads QC'ed and trimmed, if they were trimmed. What was the read length? How many reads in total were sequenced? In the annotation section, "gap filling via", what does that really mean? What gaps? Do you mean large regions of the genome without RNASeq reads mapping to them? Or do you mean missing exons in genes with low sequencing depth? And remind me, what to expect for males and females in a ZW system. In XY animals, there are a large number of male specific genes that wouldn't be transcribed. Is that true of ZW systems too? Or maybe, given biodiversity, is that true of this particular system?

 For PacBio, what was the read N50 and the total number of subreads generated? 

 The methods do not describe how the pseudomolecules were made. Presumably it was using Hi-C data, but looking at Figure 4 there are some regions that seem like they would have been difficult to distinguish. (Maybe if I knew where the scaffold boundaries are it would be more clear). But also, when you know you have two scaffolds that belong in a pseudomoleucle, how were they put together. How were they both ordered and oriented? Is there a way that if I wanted to I could now disentangle the two scaffolds and differentiate scaffold breaks from pseudomolecule breaks?

 I know, everyone loves BUSCO. But that's just ~200 proteins when you expect 12,000. I would love it if you could generate a promer plot of your pseudomolecules against a closely related Lepidoptera (particularly if there is one that has closed chromosomes). It really would give a bigger picture of the completeness of your genome. It would also show if the genomes are colinear which might help identify if there are any misassemblies. (I expect that intrachromosome rearrangements >>> interchromosome rearrangements when I say this, although not sure if that is true in Leipidoptera). You might think it is beyond the scope of the announcement. But it really is SO easy to do and would say SO much with respect to the quality of the assembly (at least more so than Figures 2-4...)

 Unless the journal requires this format, I would prefer that the WGS accession and the SRA accessions (either the experiment or the run accessions) are in the data availability section rather than the table. Or that they are both places.  In addition, annotation is described and I'm not finding that anywhere. It very well could be that I'm incompetent, but I checked several different places associated with this accession  (CAJHVI010000000 as well as GCA_905147385.1) and I don't see them. Even when all else fails, usually you can see the genes in the graphical viewer at NCBI, but I don't see them. Please ensure the annotation was deposited and is available. 

 I know insects sometimes have HGT/LGT from Wolbachia. The announcement doesn't mention this. I think it is important for all genome announcement of arthropods (and maybe nematodes) to address this issue.  Were all Wolbachia reads screened out, or were they left in? Are there integrations? If there are integrations, how were they handled with assembly? How were they handled for annotation?  

 Lastly, did this female have a Wolbachia endosymbiont or any other endosymbiont? Are they known to have endosymbionts? Has that even been assessed? Was there evidence for any endosymbionts in the genome data? Would the presence of endosymbionts expect to vary based on the tissues used for each of these extractions?

Are sufficient details of methods and materials provided to allow replication by others?

Partly

Is the rationale for creating the dataset(s) clearly described?

Yes

Are the datasets clearly presented in a useable and accessible format?

Partly

Are the protocols appropriate and is the work technically sound?

Yes

Reviewer Expertise:

genomics of host-microbe interactions, including insect-endosymbiont systems

I confirm that I have read this submission and believe that I have an appropriate level of expertise to confirm that it is of an acceptable scientific standard, however I have significant reservations, as outlined above.
